# Loss of the Synaptic Vesicle Protein SV2B Results in Reduced Neurotransmission and Altered Synaptic Vesicle Protein Expression in the Retina

**DOI:** 10.1371/journal.pone.0005230

**Published:** 2009-04-17

**Authors:** Catherine W. Morgans, Patricia Kensel-Hammes, James B. Hurley, Kimberly Burton, Rejean Idzerda, G. Stanley McKnight, Sandra M. Bajjalieh

**Affiliations:** 1 Department of Pharmacology, University of Washington, Seattle, Washington, United States of America; 2 Department of Biochemistry, University of Washington, Seattle, Washington, United States of America; 3 Casey Eye Institute, Oregon Health & Sciences University, Portland, Oregon, United States of America; University of California, Berkeley, United States of America

## Abstract

The Synaptic Vesicle Protein 2 (SV2) family of transporter-like proteins is expressed exclusively in vesicles that undergo calcium-regulated exocytosis. Of the three isoforms expressed in mammals, SV2B is the most divergent. Here we report studies of SV2B location and function in the retina. Immunolabeling studies revealed that SV2B is detected in rod photoreceptor synaptic terminals where it is the primary isoform. In mice lacking SV2B, synaptic transmission at the synapse between photoreceptors and bipolar neurons was decreased, as evidenced by a significant reduction in the amplitude of the b-wave in electroretinogram recordings. Quantitative immunoblot analyses of whole eyes revealed that loss of SV2B was associated with reduced levels of synaptic vesicle proteins including synaptotagmin, VAMP, synaptophysin and the vesicular glutamate transporter V-GLUT1. Immunolabeling studies revealed that SV2B is detected in rod photoreceptor synaptic terminals where it is the primary isoform. Thus, SV2B contributes to the modulation of synaptic vesicle exocytosis and plays a significant role in regulating synaptic protein content.

## Introduction

The secretion of neurotransmitter can be distinguished from other forms of exocytosis by its strict dependence on calcium, speed, and plasticity. These specialized features are conferred by proteins unique to regulated secretion, including some isoforms of synaptotagmin, complexin, cytomatrix proteins (reviewed in [1], [2]), and Synaptic Vesicle Protein 2 (SV2). All of these proteins are members of multi-gene families with isoforms that are co-expressed to varying degrees in different neuronal cells. Differences in isoform action, expression, and localization are hypothesized to contribute to differences in neuronal and endocrine cell functioning.

SV2 is a gene family that consists of three highly related membrane glycoproteins in mammals (SV2A, SV2B, SV2C) [3], [4], [5], [6]. Of the three isoforms, SV2B is the most divergent. In contrast to SV2A and SV2C, SV2B lacks significant portions of the cytoplasmic amino terminus, which mediates protein interactions in SV2A and SV2C [7]. SV2B also lacks regions of the large lumenal domain between transmembrane domains 7 and 8 that are present in SV2A and SV2C [5]. SV2B mRNA expression shows developmental variation, being more broadly expressed in neonatal brain than adult brain consistent with it playing a role in synapse development [8]. Finally, SV2B has been reported to be the exclusive SV2 isoform in ribbon synapses in retina and the pineal gland [9], [10]. Together these observations support the idea that SV2B may function differently in the synapse than other SV2 isoforms. On the other hand, loss of SV2B does not appear to affect neurotransmission in neurons that also express SV2A, which is consistent with the two isoforms performing an identical function, or SV2B performing only a subset of functions performed by SV2A [11], [12].

To identify the role of SV2B at the synapse, we examined the effects of SV2B gene disruption on neurotransmission in retina, thereby taking advantage of the reported absence of SV2A in ribbon synapses.

## Methods

### Antibodies

Anti-Glyceraldehyde-3-phosphate dehydrogenase, (GAPDH) mAb was from CalbioChem. Anti- Synaptophysin, p38 mAb and anti-Vesicular glutamate transporter 1, Vglut1 mAb were from Millipore. The anti-synaptophysin antibody produces no labeling in tissue from synaptophysin knockout mice [13]. The anti-vglu1 antibody antibody produces no labeling of samples from Vglut1 knockout mice [14]. Anti-Vesicle associated membrane protein 2, Vamp2 mAb and the anti-SV2B used for immunolabeling were from Synaptic Systems. The antibody against VAMP2 does not label tissue from VAMP-2 knockout mice [15]. Anti-SV2B used in Western analyses does not label cells not expressing SV2 B (here and [8]). Anti-SV2 mAb [16] and anti-synaptotagmin mAb M48 [17] were generated from cells provided by Dr. R. Kelly. Anti-SV2A pAb was generated against the first 20 residues of rat SV2A . This antibody does not label cells that do not express SV2A [8] and produces no labeling of brain homogenates from SV2A knockout mice [18]. Anti-synaptotagmin pAb was generated against the cytoplasmic domain of rat synaptotagmin 1 [19].

### Generation of SV2B minus mice

**Figure 1:**
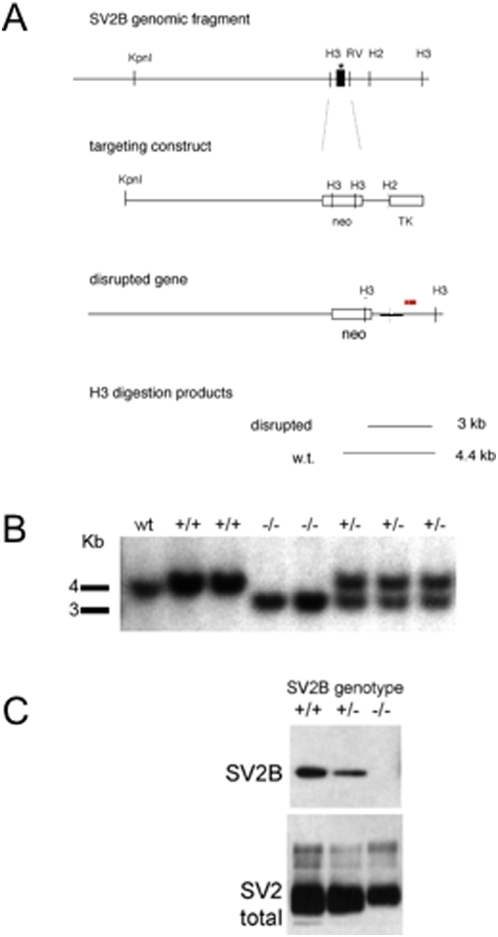
Generation of SV2B knockouts. A) Targeting construct. A region of the SV2B gene containing the first exon (depicted as a black box) was cloned from a mouse 129/SVJ genomic library. The genomic fragment was used to generate the indicated targeting vector in which exon 1 was replaced with a gene encoding neomycin resistance. A thymidine kinase gene, which makes cells sensitive to gancyclovir was placed at the end of the targeting construct to allow for negative selection of non-homologous recombination events. The mutant gene produces a smaller HindIII (H3) restriction fragment as detected in Southern analyses with a probe indicated by the band above the disrupted gene. Notation key: H2 = HindII restriction site, H3 = HindIII restriction site, RV = EcoRV restriction site, neo = DNA encoding neomycin resistance, TK = DNA encoding thymidine kinase. B) Southern blot of Hind III-digested genomic DNA from littermates resulting from the crossing of heterozygous breeders demonstrating the production of mice homozygous for the SV2B gene disruption. C) SV2 expression in brain of SV2B mutants. 25 ug of a Triton X-100 extract of whole brain was probed with antibodies to the indicated protein. Antibody binding was visualized with HRP-conjugated secondary antibody reacted with enhanced chemiluminescence (ECL) reagent and exposed to film. Disruption of the SV2B gene results in loss of full length SV2B and a decrease in total SV2. Data are representative of experiments from two series of mice.

A portion of the SV2B gene was isolated from a mouse 129SV genomic library (Stratagene) by screening with a PCR-generated probe encoding bases 60–245 of the rat SV2B cDNA. A fragment of approximately 7.5 kb was subcloned from the genomic library. This fragment contained the exon encoding the translation start site through most of the first transmembrane domain of the SV2A cDNA. A targeting construct was generated in which this exon and surrounding DNA were replaced with a gene encoding neomycin phosphotransferase as illustrated in Figure 1. DNA encoding thymidine kinase was placed at the end of the short arm of the targeting construct to allow for negative selection against non-homologous recombination using the antiviral agent gancyclovir.

Embryonic stem cells were grown on feeder layers of mouse embryonic fibroblasts, generated from mice transgenic for the neomycin resistance gene that had been pre-treated with mitomycin C to prevent proliferation. Cells were grown in DMEM with 15% fetal calf serum, penicillin/streptomycin, non-essential amino acids, and leukemia inhibitory factor. ES cells were transfected at a density of 1.2×10^7^ cells/ml with 25 ug/ml linearized targeting construct by electroporation. Cells were placed under selection in G418 (Gibco/BRL) and Gancyclovir (a gift from Syntex Corporation). Resistant colonies were screened for homologous recombination by Southern analysis of HindIII digested genomic DNA using the probe depicted in Figure 1.

Three cell lines carrying the SV2B disruption were injected into C57BL/6 blastocysts and implanted into pseudopregnant females. Pups were evaluated for ES cell incorporation by coat color. One of five chimeric males produced offspring heterozygous for the SV2B gene disruption. These offspring were bred with each other to produce animals homozygous for the mutation. A line of genetically matched wild-type mice was generated from the wild-type offspring of heterozygote (SV2B+/−) crosses and used as control animals. Unless indicated, studies were conducted with animals that were, on average 50% 129/SVJ and 50% C57BL/6. All animals used in these studies were genotyped by Southern analysis of DNA isolated from tail clippings as indicated in Figure 1.

### Immunohistochemistry

Immunolabeling of retina sections was performed as described previously [20], [21]. Briefly, mouse eyecups were prepared from freshly dissected eyes. Eyecups were fixed by immersion in freshly prepared, ice cold 4% (w/v) paraformaldehyde in phosphate buffer (0.1 M, pH 7.4) for 20 minutes, washed in phosphate buffer (0.01 M, Ph 7.4) and then cryoprotected by consecutive immersion in cold 10% and 20% sucrose in phosphate buffered saline (PBS) for 1–2 hrs each, then transferred to vials of 30% sucrose and left overnight at 4°C. The tissue was embedded in OCT (Sakura Finetek, Torrence, CA), frozen, and cut at 15 µm thickness on a cryostat. Sections were mounted onto Super-Frost glass slides, air-dried and stored at −80°C until used for staining. After thawing to room temperature (RT), eyecup sections were blocked by incubation at RT for 30–60 min in antibody incubation solution [AIS: 3% (v/v) normal horse serum, ±0.5% (v/v) Triton X-100, 0.025% (w/v) NaN3 in PBS]. The sections were then incubated with primary antibody diluted in AIS for either 1–2 h at RT or at 4°C overnight. Mouse retina sections from SV2B knockout and wild-type littermates were labeled with primary antibodies against SV2 (1∶200), SV2A (1∶1000), SV2B (1∶100), vGlut1 (1∶1000), synaptophysin (1∶500), ribeye (ctbp2; 1∶5000; BD Transduction Laboratories), beta-2 calcium channel subunit (1∶100; Chemicon International), Cav1.4 (1∶20; Morgans et al., 2005), and synaptotagmin (1∶1000). Secondary antibodies coupled to either CY3 (Jackson ImmunoResearch Laboratories, West Grove, PA), Alexa488, or Alexa594 (Invitrogen) were used at dilutions of 1∶500 to 1∶2000. For some antigens (synaptotagmin, SV2A and SV2B), peanut agglutinin-Alexa488 was added to Alexa594-coupled secondary antibody solutions at a 1∶1000 dilution in order to visualize cone terminals. Images were acquired at a resolution of 1024×1024 on an Olympus Fluoview1000 confocal imaging system with a 60×/1.42 oil immersion objective. All confocal figures show single optical sections of <1 µm thickness. For figures, Adobe Photoshop 7.0 was used to adjust brightness and contrast uniformly across the image with identical adjustments made to pairs of knockout and wild-type images.

### Analyses of protein expression

Protein expression was determined by immunoblot analysis of Triton X-100 extracts or post-nuclear supernatants of whole brain obtained from littermate pups of heterozygote crosses. To assess expression in eyes, eyes were pooled and homogenized in phosphate buffered saline after which Triton X-100 was added to a final concentration of 1%. The mixture was extracted 1–2 hours with gentle agitation after which insoluble material was removed by centrifugation at 19,000× g for 30 min. To assess expression in brain, post-nuclear supernatants of brain were made by homogenizing brains in 20 mM Hepes pH 7.5, 250 mM sucrose, 2 mM MgCl_2_ and centrifuging at 1300× g for 5 minutes. Protein concentration was assayed using the Biorad Coomasie-based protein assay with bovine serum albumin as a standard. Equal amounts of protein from wild-type, heterozygous and homozygous littermates were separated on 12.5% SDS-PAGE gels and transferred to PVDF (0.45um, Millipore) in 20% MeOH, 25mM Tris, 192 mM Glycine. Transfer was done at 100 volts for 1 hr at 4°C. Blots were stained with Ponceau Red to verify equivalent loading across lanes. Blots were blocked 5% milk in phosphate buffered saline with 0.025% Tween 20. Incubations with the primary antibody were done inblocking buffer, either for 1 hour at RT or O/N at 4°C with agitation. Blots were washed a minimum of 3×15 min in phosphate buffered saline with 0.025% Tween 20 and then incubated for 1–2 hr at rt in HRP-conjugated secondary antibodies diluted in the blocking buffer. Binding of the HRP-conjugated secondary antibody was detected with SuperSignal West Dura reagent (Pierce/Thermo), and the images were acquired and analyzed using a Kodak Image Station 440 and analyzed with Kodak software.

*ERG analyses were* performed as previously described [22]. Briefly, 5–6 week old mice were dark-adapted overnight and anaesthetized with intraperitoneal injection of Ketamine and Xylazine (140 mg/kg and 0.5 mg/kg body weight respectively). Pupils were dilated by topical application of tropicamide and phenylephrine 15–20 minutes before any ERGs were recorded. Mice were maintained at 37°C throughout the recording sessions. All manipulations were done under infrared illumination. Flashes were delivered from a photographic flash unit focused on the eye through a fiber optic cable and lens. A gold ring electrode embedded in a contact lens (Bayer, 2000) was placed on a drop of 2–3% methylcellulose on the cornea and a copper reference electrode was placed in the mouth. ERG signals were amplified 10,000×, filtered between 1 Hz to 3 kHz and sampled at 5 kHz. Responses to test flashes from dim to moderate intensity caused 9, 90, 900, 9000, and 90,000 photoisomerisations per rod in dark adapted wild-type mice. Littermate pairs resulting from SV2B +/− crosses were used at a minimum of 4 weeks of age. Both 50% 129/SVJ and 50%, C57BL/6 and 99.9% C57BL/6 (F12) genetic backgrounds were used.

## Results

### Targeted disruption of the SV2B gene

A portion of the SV2B gene containing a single exon encoding the translation start site through the beginning of transmembrane domain 2 was isolated from a mouse 129/SVJ genomic library as described under *Experimental Procedures*. This genomic fragment was incorporated into the targeting construct depicted in **Figure 1A** and was used to generate mice homozygous for the SV2B gene disruption (**Figure 1B**). Animals homozygous for the SV2B gene disruption constitute approximately one quarter of live births resulting from the mating of heterozygous mutants and are viable and fertile. This indicates that SV2B is not essential for survival and contrasts with loss of SV2A, which generally results in death within 21 days of birth [12], [18].

Brain homogenates from SV2B −/− mice lacked detectable SV2B protein as detected by immunoblot (western) analysis (**Figure 1C**), indicating that full-length SV2B protein is absent and thus confirming disruption of expression. Total SV2, detected with a monoclonal antibody that recognizes all known isoforms [16], was also reduced in the brain of SV2B knockouts as expected. However, this reduction was less than observed in SV2A knockouts [18], indicating that SV2B is not the primary SV2 isoform in brain (**Figure 1C**). Northern analysis of RNA isolated from the brains of SV2B knockout homozygotes revealed the absence of full-length SV2B mRNA, though a smaller RNA hybridized to SV2B cDNA probes (data not shown). Because all available anti-SV2B antibodies are directed against the amino terminus of SV2B, we cannot rule out the possibility that a truncated SV2B protein is expressed. We note, however, that the next possible codon for methionine that would produce an in-frame message is in the region encoding the cytoplasmic domain preceding transmembrane domain 6. Given that many point mutations in SV2 lead to improper protein trafficking ([23] and our unpublished observations), it is highly unlikely that a truncated SV2B is expressed in synapses. We cannot entirely rule out the possibility, however, that a truncated protein is contributing to the effects we observe.

### SV2B is the primary but not exclusive SV2 isoform in rod photoreceptor synapses

**Figure 2:**
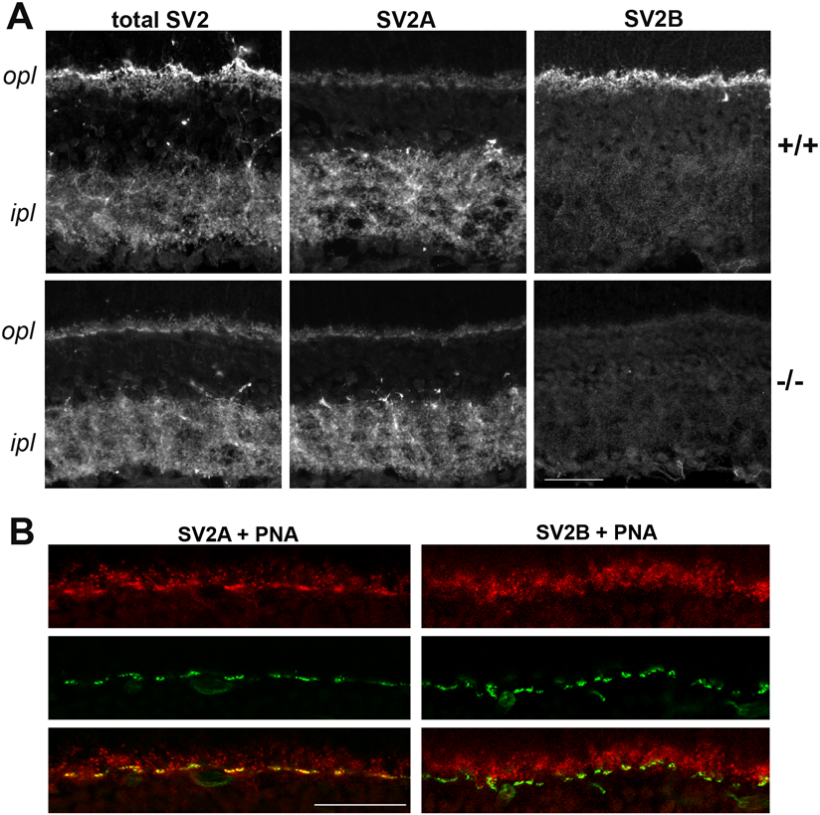
SV2A and SV2B are differentially distributed in photoreceptor terminals. A) SV2A is expressed in both inner and outer plexiform layers whereas SV2B is expressed primarily in the outer plexiform layer. Retina sections from SV2B knockout (−/−) and wild-type (+/+) mice were labeled by fluorescence with antibodies recognizing all SV2 subtypes (total SV2), or antibodies specific for either SV2A or SV2B. B) SV2A is the predominant SV2 isoform in cone terminals and SV2B predominates in rod terminals. The outer plexiform layer is shown for wild-type retina sections double labeled for peanut agglutinin (PNA) conjugated to Alexa-488 (green) and either SV2A or SV2B (red). Areas of overlap between the two fluorophores appear yellow. Both anti-SV2A and anti-SV2B labeled PNA-negative synapses, consistent with both isoforms being present in rod photoreceptor terminals. Abbreviations: opl, outer plexiform layer; ipl, inner plexiform layer. The scale bars represent 30 µm.

We examined SV2A, SV2B and total SV2 in retina from wild-type and SV2B knockout mice using isoform-specific antibodies and an antibody that recognizes all three isoforms. SV2B was previously reported to be the sole SV2 isoform in rod photoreceptor synapses [9] and we therefore anticipated that these synapses would lack all SV2 in SV2B knockouts. We compared the presence of SV2A and SV2B to the cone marker peanut agglutinin (PNA) in retina from wild-type and SV2B knockout mice. As previously reported, we observed strong SV2A immunoreactivity in PNA-positive cone terminals, and SV2B immunoreactivity primarily in rod (PNA-negative) terminals in the outer plexiform synaptic layer (OPL) of wild-type mice, (**Figure 2**). In contrast to the findings of Wang et al. (2003), however, we observed faint labeling of PNA-negative synapses with anti-SV2A, indicating that SV2A is expressed in rod synapses. This labeling of rod terminals is unlikely to be a fixation artifact, since the sections were lightly fixed and labeling was specific to synaptic terminals. Also in contrast to the findings of Wang et al. (2003), we saw little SV2B labeling in the inner plexiform layer, consistent with SV2B being expressed primarily in rod photoreceptor terminals.

Total SV2 was significantly reduced in the OPL of retina from SV2B knockout mice, where the labeling pattern was indistinguishable from anti-SV2A labeling (**Figure 2**). These results support the conclusion that SV2B is the primary, though not exclusive, SV2 isoform in rod photoreceptor synapses. Thus both our results and those of Wang et al. support the conclusion that SV2B is the primary SV2 isoform found at rod synapses.

### Loss of SV2B leads to reduced neurotransmission in the ribbon synapses between photoreceptors and bipolar neurons of the retina

**Figure 3:**
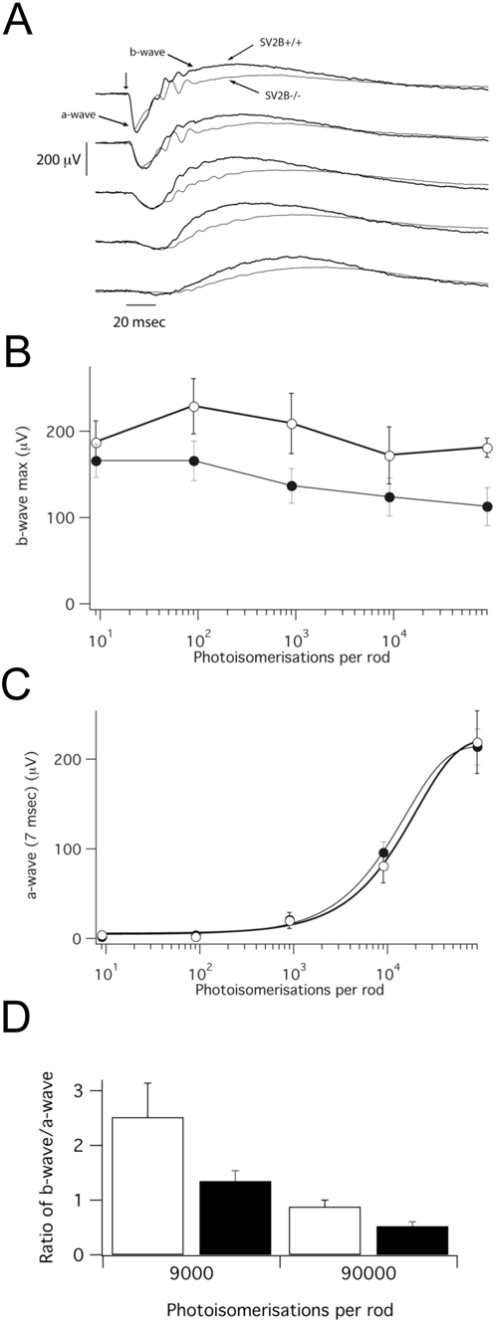
Loss of SV2B results in reduced neurotransmission between photoreceptors and bipolar neurons of the retina. A) Average ERG traces from wild-type and SV2B−/− retina in response to stimuli of (top to bottom) 90,000, 9,000, 900, 90 and 9 photo-isomerizations per rod per flash. (n = 5 wt, 8 SV2B−/− mice). B) Average b-wave amplitudes at different light intensities showing average intensity decrease in mice from SV2B knockouts (n = 5 wt, 8 SV2B−/− mice). Error bars represent S.E.M. C) Average a-wave amplitudes showing no difference in amplitude in wt and SV2B knockouts. D) The ratio of b- to a-wave amplitude at the two strongest stimulus intensities was ∼50% lower in SV2B−/− mice (p = 0.015 for 90,000, p = 0.026 for 9000, students t-test).

To explore the function of SV2B, we compared neurotransmission in the retinas of wild-type and SV2B knockout mice by measuring the electroretinographic response to light flashes of varying intensity [22]. This procedure measures electric potential flux in the retina. The amplitudes of the a-wave component of the measure, which reflects closure of cGMP-gated ion channels in the outer segment membrane, were not significantly different in SV2B−/− retinas (**Figure 3C**), indicating that the channels regulating membrane conductance in photoreceptors function normally in the absence of SV2B. In contrast, b-wave amplitudes, which reflect synaptic transmission between photoreceptors and bipolar neurons, were reduced ∼30% in retinas of SV2B−/− mice compared to SV2B+/+ mice (**Figure 3A–B**). Decreased b-wave amplitudes were observed at stimulus intensities in which only rod photoreceptors were activated (9 and 900 photoisomerisations/sec) and those in which both rods and cones were activated (9000 and 90,000 photoisomerisations/sec) (**Table 1**). When b-wave amplitudes were normalized by calculating the b/a wave ratio, we observed that values from SV2B knockouts were ∼50% lower at the two highest stimulus intensities (**Figure 3D**). Examination of ribbon synapses in retinas from wild-type and SV2B −/− mice did not reveal obvious differences in photoreceptor synapse ultrastructure (Supplemental Figure S1). This contrasts with the striking change in ribbon morphology observed in Bassoon knockout mice, which demonstrate a similar neurotransmission phenotype [24]. Thus diminished transmission between photoreceptors and bipolar neurons does not appear to be due to gross changes in ribbon synapse development. Taken together, these observations indicate that SV2B, like SV2A [18], [25], is a positive modulator of synaptic transmission.

**Table 1:** ERG b-wave amplitudes are decreased in the retina of mice lacking SV2B.

R* per rod per flash	SV2B+/+	SV2B−/−	KO/wt ratio	*p* (t-test)
90,000	181 +/− 11 (n = 4)	113 +/− 22 (n = 8)	0.62	0.065
9,000	172 +/− 33 (n = 5)	124 +/− 22 (n = 8)	0.72	0.114
900	209 +/− 35 (n = 5)	137 +/− 20 (n = 8)	0.65	0.038
90	229 +/− 32 (n = 5)	166 +/− 23 (n = 8)	0.72	0.065
9	187 +/− 25 (n = 5)	166 +/− 19 (n = 8)	0.89	0.258

Mean hyperpolarization amplitudes +/− S.E.M. in response to light flashes of the indicated intensity.

### Levels of several synaptic vesicle proteins are reduced in eyes of SV2B knockouts

All SV2 isoforms bind to the synaptic vesicle protein synaptotagmin [7], [19], [26], and loss of SV2B has been reported to produce a selective loss of synaptotagmin 1 in the outer plexiform synaptic layer of the retina [26]. To quantify changes in synaptic vesicle protein expression, we compared the levels of synaptotagmin and other synaptic vesicle proteins in eyes of SV2B wild-type and knockout mice by quantitative Western analysis. Eyes from multiple mice of each genotype were pooled and homogenates probed with antibodies directed against the synaptic vesicle proteins SV2 (total), SV2A, SV2B, synaptotagmin, synaptophysin, the vesicular glutamate transporter V-glut1, and VAMP. The amount of protein loaded was adjusted for each antibody so that signal intensity fell within the linear range of the assay (Supplemental Figure S2). Samples from each genotype were run multiple times in each blot to control for variability. The average value for SV2B−/− eyes was expressed as the percent of the average value for SV2B+/+ eyes from the same blot. These analyses revealed decreased levels of synaptotagmin, synaptophysin, V-Glut-1, and VAMP in eyes from SV2B−/− mice (**Table 2**). Levels of SV2A and GAPDH (which is found on synaptic vesicles but is also present in all cells of the eye) did not change with the loss of SV2B. Since the retina is the major site of synapses in the eye, these data indicate that multiple synaptic vesicle proteins are reduced in retinal synapses in the absence of SV2B. While the reduction in protein expression we observed may seem large given the localized expression of SV2B in the retina, we note that photoreceptor synaptic terminals contain hundreds of thousands of synaptic vesicles per synapse. This contrasts with the tens to hundreds of vesicles in the synapses of the inner retina. It is therefore likely that, although there are many more synapses in the inner retina, the pool of photoreceptor synaptic vesicles represents the majority of retinal synaptic vesicles.

**Table 2:** Loss of SV2B decreases the expression of several synaptic vesicle proteins.

Protein	% wild-type
SV2B	0.3 +/−0
SV2A	98 +/− 5
SV2 (total)	57 +/− 13
Synaptotagmin (mAb)	61 +/− 4
Synaptotagmin (pAb)	44 +/− 8
Synaptophysin	55 +/− 5
VAMP2	71 +/− 5
V-glut1	66 +/− 4
GAPDH	102 +/−7

Eyes from two SV2B+/+ and two age-matched SV2B−/− mice were pooled and extracted after which equal amounts of protein processed for quantitative Western analysis as described under *Methods*. Each sample was run six times on a minimum of three blots. The ratio of average values (KO/wt) was calculated for each blot and the ratios averaged across blots. All proteins except SV2A and GAPDH were reduced compared to wild-type levels. Results are representative of another study done with eyes pooled from 4 mice of each genotype.

**Figure 4:**
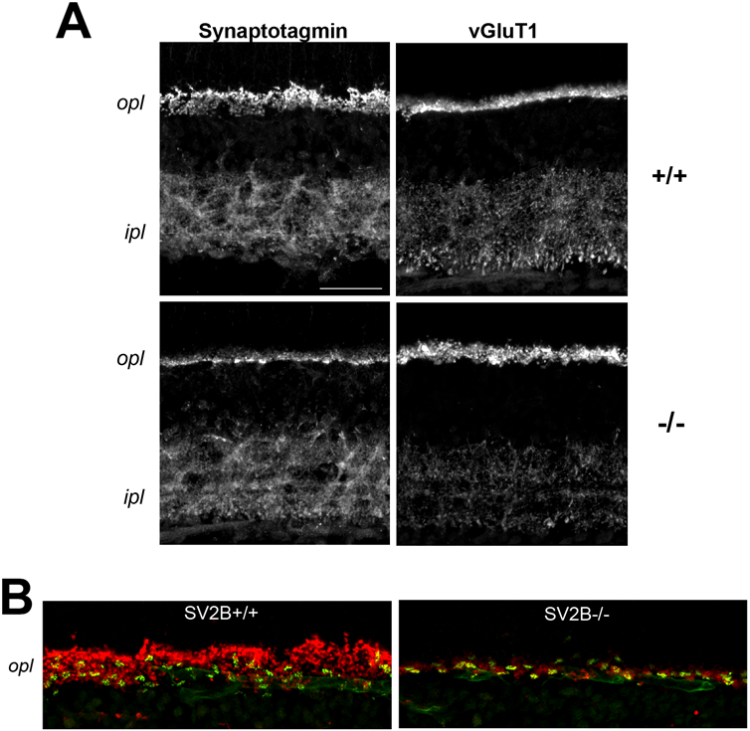
Distribution of synaptic vesicle proteins in SV2B knockout (−/−) and wild-type (+/+) retina. A) Synaptotagmin labeling is markedly reduced in the OPL of the −/− retina. Retina sections from SV2B −/− and +/+ mice were labeled by immunofluorescence with antibodies against synaptotagmin or vGluT1. Negligible differences were observed for vGluT1 in the OPL between the −/− and +/+ retina sections, whereas synaptotagmin labeling was visibly reduced in the OPL. B) Synaptotagmin in OPL synapses of SV2B −/− retina is primarily associated with cone terminals. The OPL of SV2B −/− and +/+ mice was double labeled for synaptotagmin (red) and the cone marker, PNA-Alexa 488 (green). A small amount of synaptotagmin labeling remains in PNA-negative synapses in the SV2B −/− OPL; however, the majority of the remaining labeling is associated with cone terminals, suggesting that synaptotagmin levels are significantly reduced in rod terminals. Abbreviations: opl, outer plexiform layer; ipl, inner plexiform layer. The scale bars represent 30 µm.

While the quantitative western blotting data point to a general decrease in synaptic vesicle proteins in the eye they do not indicate whether different types of synapses are differentially affected. To assess the location of the changes in protein expression, we probed retina sections with antibodies to multiple synaptic proteins. Immunolabeling with antibodies that recognize synaptotagmin 1 and 2 revealed a reduction, though not a complete absence, of synaptotagmin labeling in the outer plexiform layer of SV2B−/− mice, particularly in rod terminals (**Figure 4**). This is in agreement with the findings of Lazzell et al (D. R. Lazzell et al., 2004) and is consistent with the interpretation that SV2B plays a crucial role in synaptotagmin trafficking or stability in rod photoreceptor synapses. We also saw a slight reduction in VGLUT1 labeling of putative rod bipolar cell terminals in the inner IPL (Fig. 4A), whereas there was no apparent decrease in other synaptic proteins including synaptophysin, synaptic ribbons (ribeye) and the presynaptic calcium channel proteins, Cav1.4 and β2 subunits (data not shown). This may be due to the non-quantitative nature of the immunolabeling approach, or to a generalized reduction of other synaptic proteins in the absence of SV2B. What our immunolabeling studies do show is that the decrease of synaptotagmin expression in the retina of mice lacking SV2B is limited to rod photoreceptor terminals in the OPL.

## Discussion

The findings presented here indicate that loss of SV2B reduces neurotransmission at rod photoreceptor synapses. This is the only physiological phenotype identified to date in SV2B knockout mice and contrasts with the normal neurotransmission observed in CNS synapses of these mice [11], [12]. The decrease is unlikely to be due to effects on synapse formation, as the gross morphology of synapses was normal. This contrasts to the “floating ribbon” phenotype seen in Bassoon knockouts [24], or the structural abnormalities obvious in electron micrographs of Gbeta5 knockouts [27], both of which produce similar effects on the ERG. Our results demonstrate that, like SV2A, SV2B acts as a positive modulator of neurotransmission, consistent with the conclusion that all SV2 proteins perform a similar function in regulating presynaptic exocytosis. Thus SV2B is likely to represent a target for therapeutic intervention analogous to SV2A, which is the target of the anti-epileptic drug levetiracetam [28].

We also found that loss of SV2B alters synaptic protein expression in the eye. Previous studies of retinal protein expression in SV2B knockouts revealed a selective loss of synaptotagmin 1 in the outer plexiform layer (OPL) of the retina [26], which the authors attributed to a selective interaction between SV2B and synaptotagmin. While we confirmed the effect on synaptotagmin, quantitative Western analyses to revealed that loss of SV2B also affects the levels of synaptophysin, VAMP and V-GLUT1. Thus SV2B has a broader effect on the levels of synaptic vesicle protein expression than was previously demonstrated. In a series of (non-quantative) anti-synaptophysin immunoblots we observed that samples from SV2B−/− mice showed a reduction in bands that migrated at the molecular weight of VAMP-synaptophysin complexes [29], [30] (data not shown). This suggests that loss of SV2 may affect the ability of VAMP to form protein complexes, an interpretation consistent with the finding that loss of the SV2A gene results in a reduction in assembled (SDS-resistant) SNARE complexes [25].

The presence of SV2A in rod photoreceptor presynaptic terminals, coupled with the observation that the decrease in synaptotagmin labeling in the OPL of SV2B−/− mice correlates well with the decrease in total SV2 immunolabeling suggests that the remaining synaptotagmin is being stabilized by SV2A. Thus it is likely that all SV2 isoforms contribute to synaptic vesicle protein composition. Our finding, that loss of SV2B affects the levels of multiple synaptic proteins, is consistent with a role for SV2 in endocytosis. SV2s contain one or more tyrosine-based endocytosis motifs that can affect the ability of the clathrin adaptor protein AP2 to bind to synaptic membranes, in part by affecting AP2 binding to synaptotagmin [31]. Thus, SV2 could influence the trafficking of proteins to synaptic vesicles by regulating clathrin-mediated endocytosis of vesicle proteins. Alternatively, SV2 might regulate the trafficking of vesicle membrane proteins from the ER/Golgi. We note with interest that the transporter-like protein, unc93b, is essential for proper trafficking of a subset of Toll receptors, which are type 1 membrane proteins like several synaptic vesicle proteins [32], [33]. In this regard, SV2 might affect the conformation and stability of vesicle membrane proteins by directly interacting with them and affecting their trafficking or folding.

If SV2 plays a major role in vesicle assembly, the neurotransmission deficit we observed could result entirely from altered vesicle protein composition or decreased vesicle formation. Since we did not observe a gross decrease in the number of vesicles in photoreceptor synapses - or in central synapses in SV2A knockouts [18] - SV2 does not appear to contribute to vesicle formation *per se*. Future work on the roles of SV2 in vesicle priming and protein trafficking will address whether it plays multiple roles at the synapse. What the findings presented here make clear is that SV2B influences the composition of synaptic vesicles. Thus, SV2 proteins may play a structural role in vesicle biogenesis, a process essential to normal neurotransmission.

## Supporting Information

Figure S1 Representative electron microscopic images of retina from two SV2B wild-type and two SV2B knockout (−/−) mice. Arrows point to ribbon synapses. *Electron Microscopic Analyses*: Retinas were dissected from eyes of two wild-type and two SV2B knockout mice and fixed in 2% paraformaldehyde, 2.5% glutaraldehyde, 0.1M Cacodylate pH 7.3, 0.1% CaCl2 overnight at 4°C. Tissue was washed 3× over 30' with 0.1M Cacodylate, pH 7.3, 0.1% CaCl2, post fixed in 1% OsO4, 1% Potassium Ferrocyanide, 0.1M Na Cacodylate pH 7.3, 1 hr on ice, and washed in double distilled water (ddH_2_O) 3× and stored overnight at 4°C. Tissue was stained with 2% aqueous Uranyl Acetate (1 hr, rt), washed in ddH2O and dehydrated in a graded series of ethanol washes. Dehydrated blocks were incubated in Propylene Oxide, 2× 10 min and infiltrated with Spurrs's Low Viscosity Epoxy. Thin sections of embedded tissue (90–100 nm) were stained with 7% UA and Reynolds Lead Citrate. Images were collected at 60,000× and 75,000× magnification by a specialist blind to mouse genotype. Electron microscopy was performed at the University of Washington Department of Pathology EM Facility
(247.34 MB PPT)

Figure S2 Calibration blots and Western data. Shown are blots and graphs of the s ignal intensity obtained from increasing amounts of postnuclear supernatant from wild-type eye probed with the indicated antibody. Also shown are representative quantitative blots showing wild-type and SV2B KO sample replicates.
(0.37 MB PDF)
