# Supplementary material for: Loss of the Synaptic Vesicle Protein SV2B Results in Reduced Neurotransmission and Altered Synaptic Vesicle Protein Expression in the Retina
Source: PLoS One. 2009 Apr 17;4(4):e5230. doi: 10.1371/journal.pone.0005230 (PMC2667261; doi:10.1371/journal.pone.0005230)
Supplement: Figure S2 — Calibration blots and Western data. Shown are blots and graphs of the s ignal intensity obtained from increasing amounts of postnuclear supernatant from wild-type eye probed with the indicated antibody. Also shown are representative quantitative blots showing wild-type and SV2B KO sample replicates. (0.37 MB PDF) [file pone.0005230.s002.pdf]

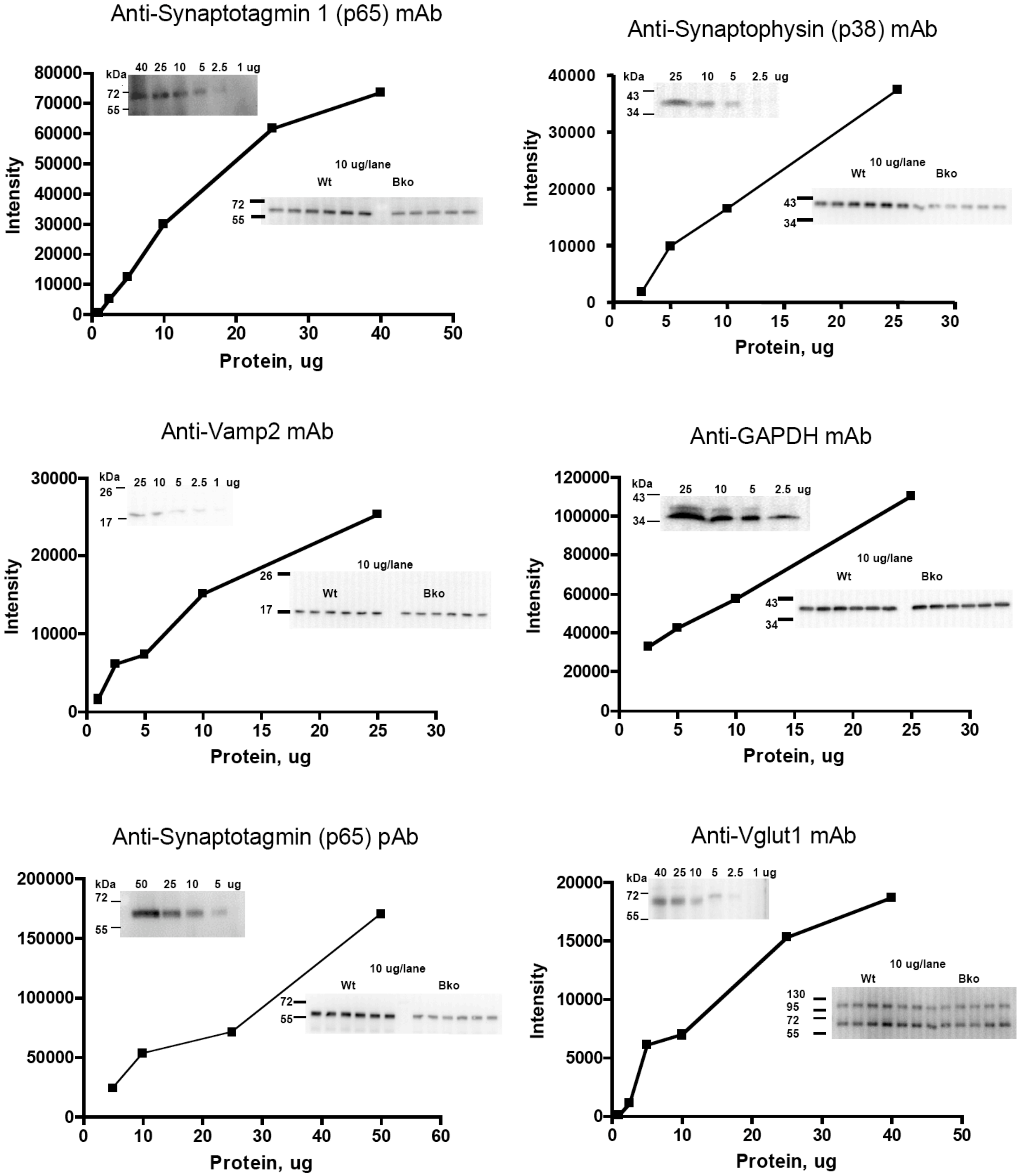

Supplemental Figure 1: Calibration blots and Western data.
Shown are blots and graphs of the s ignal intensity obtained from increasing amounts of postnuclear supernatant from wild-type eye probed with the indicated antibody. Also shown are representative quantitative blots showing wild-type and SV2B KO sample replicates.
